# Meningitis due to inflammatory reaction to *Echinococcus* antigen after the resection of cerebral alveolar hydatid cyst

**DOI:** 10.1093/jscr/rjaf072

**Published:** 2025-02-21

**Authors:** Taishin Yoshida, Shota Yamamoto, Sayaka Yuzawa, Takuma Takano, Takahiro Sanada, Masato Saito, Mishie Tanino, Manabu Kinoshita

**Affiliations:** Department of Neurosurgery, Asahikawa Medical University, Midorigaoka-higashi 2-1-1-1, Asahikawa, Hokkaido 078-8510, Japan; Department of Neurosurgery, Osaka General Medical Center, 3-1-56 Bandai-higashi, Sumiyoshi-ku, Osaka 558-8558, Japan; Department of Diagnostic Pathology, Asahikawa Medical University Hospital, Midorigaoka-higashi 2-1-1-1, Asahikawa, Hokkaido 078-8510, Japan; Department of Neurosurgery, Sapporo Higashi Tokushukai Hospital, 3-1, Kita 33-jo Higashi 14, Higashi-ku, Sapporo, Hokkaido 065-0033, Japan; Department of Neurosurgery, Japanese Red Cross Kitami Hospital, Kita6-jo Higashi 2-1, Kitami, Hokkaido 090-8666, Japan; Department of Neurosurgery, Asahikawa Medical University, Midorigaoka-higashi 2-1-1-1, Asahikawa, Hokkaido 078-8510, Japan; Department of Diagnostic Pathology, Asahikawa Medical University Hospital, Midorigaoka-higashi 2-1-1-1, Asahikawa, Hokkaido 078-8510, Japan; Department of Neurosurgery, Asahikawa Medical University, Midorigaoka-higashi 2-1-1-1, Asahikawa, Hokkaido 078-8510, Japan

**Keywords:** meningitis, cerebral alveolar hydatid cyst, inflammatory reaction, puncture-aspiration-injection-respiration

## Abstract

Meningitis after resection of a cerebral alveolar hydatid cyst (CAHC) is rare. Although several etiologies are proposed for meningitis, including recurrence or inflammatory reaction to dead echinococcal antigens, the effectiveness of treatment strategies specifically targeting the inflammatory reaction has not been reported. A 72-year-old patient presented with a brain cyst suggestive of CAHC. The cyst ruptured during resection, which was immediately replaced with 95% ethanol to obliterate the viable larvae, followed by cyst removal. One-month post-surgery, the patient showed signs of meningitis suggestive of a recurrence of cerebral hydatidosis. However, the negative sign of the recurrence on the brain and whole spine MRI suggested an inflammatory reaction to the obliterated *Echinococcus* antigen. An increased dose of prednisone resulted in the patient’s complete recovery without recurrence for 1.8 years. Adding steroids to the anthelmintic maintenance treatment could be reasonable if the inflammatory reaction is more likely to be the cause.

## Introduction

*Echinococcosis* is a worldwide anthropozoonosis except for Antarctica [1]. Alveolar *Echinococcus* tends to parasitize in humans in the liver (75%–85%), and secondarily in the lungs (20%) [2]. However, larvae can infrequently develop in the brain, causing cyst formation.

Resection followed by systemic chemotherapy is considered first-line treatment for cerebral alveolar hydatid cyst (CAHC) [3, 4], while recurrence occasionally occurs, leading to meningitis with neurological deterioration [5–8]. Meningitis following surgical resection with cyst rupture should remind progression of the disease as a primary potential cause [3–6]. On the other hand, other possible etiologies, such as allergic or inflammatory reactions against the obliterated *Echinococcus* [5, 6] or chemical meningitis with unknown etiology [3] should also be considered.

Here, we report a case of a patient who suffered from meningitis during chemotherapy following a CAHC resection with intraoperative cyst rupture. Intraoperative cyst rupture was addressed with aspiration with instillation of 95% alcohol, referring to the puncture-aspiration-injection-respiration (PAIR) technique, originally developed for treating hepatic alveolar hydatid cyst (HAHC). Meningitis was successfully treated by an increased dose of prednisone under an estimated diagnosis of inflammatory reaction against the obliterated *Echinococcus*.

## Case report

**Figure 1:**
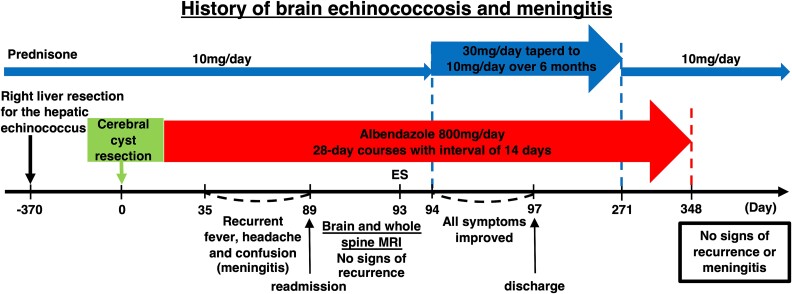
Schematic outline of the patient’s brain echinococcosis and meningitis history and sequence of treatments. ES = epileptic seizure. A flowchart of the patient’s clinical course showing the dose of prednisone on the top, the surgical procedure and the subsequent maintenance therapy in the middle, and the symptom timeline and test results on the bottom.

A 72-year-old female who underwent right liver resection for HAHC one year prior was referred to our institution for treatment against a cystic lesion in the left parietal lobe of the brain (Fig. 1). She also had a past medical history of rheumatoid arthritis, which was controlled by 10 mg of daily prednisolone.

**Figure 2:**
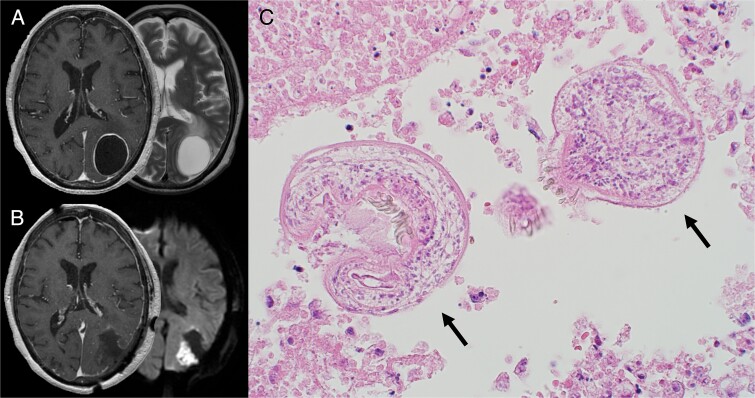
(A) Preoperative MRI. Axial contrast-enhanced T1-weighted (left) and T2-weighted MRI (right) demonstrated a ring-enhancing cystic lesion in the left parietal lobe with edema extending ventrally. (B) Postoperative MRI. Axial contrast-enhanced T1-weighted image (left) showed complete cyst resection. Diffusion-weighted image (right) showed cerebral infarction adjacent to the cyst wall caused by vascular injury during the operation. (C) HE staining of the cyst showed two protoscoleces (black arrows). Alt text: Preoperative MRI images of brain *Echinococcosis* at the top left and postoperative MRI images showing complete cyst resection at the bottom left. A microscopic image on the right is a Hematoxylin and eosin (H&E) stained specimen showing two protoscoleces.

On admission, she presented nausea with a neurological examination revealing right homonymous hemianopia, anarithmia, agraphia, and finger agnosia without lateral agnosia (incomplete Gerstmann syndrome). Gadolinium-enhanced T1-weighted magnetic resonance imaging (MRI) of the brain revealed a single cystic lesion in the left parietal lobe with a diameter of 44 mm surrounded by a contrast-enhanced smooth rim with a small daughter cyst (Fig. 2A). A mild edema was observed mainly ventral to the cyst (Fig. 2A). The patient was suspected to be suffering from CAHC based on the past medical history of HAHC.

A surgical resection of the cyst was performed to deal with the cyst’s intracranial mass effect. The cyst was found to be covered with hard, fibrous tissue. Despite extreme caution not to rupture the cyst, the strong adhesion with the arachnoid membrane of the cyst wall’s posterior region tore, and some internal fluid disseminated to the surgical field. The disseminated fluid was turbid and immediately aspirated to prevent further dissemination, followed by replacing the cyst content with 95% alcohol, referring to the PAIR technique. The cyst wall was removed entirely after aspirating the injected alcohol (Fig. 2B). Histological examination confirmed the presence of CAHC (Fig. 2C).

Hydrocortisone was given intravenously for 2 days postoperatively to prevent adrenal insufficiency from discontinuing long-term steroids. The patient was then switched back to the original 10 mg/day oral dose of prednisolone for rheumatoid arthritis and continued this regimen thereafter. Anthelmintic maintenance treatment was initiated on the day after surgery with a 28-day-on and 14-day-off administration of 800 mg of albendazole. However, the patient started to exhibit a fever of unknown origin, confusion, and generalized pain with a headache on the 27th day after the first cycle of albendazole treatment, with repeated exacerbation and remission of symptoms (Fig. 1). Furthermore, the patient exhibited a mild but sudden disturbance of consciousness on day 90, which led to the administration of an anti-epileptic drug. The cause of these symptoms was initially suspected to be local recurrence or metastasis of cerebral alveolar hydatidosis (CAH) originating from the cyst’s leaked content during the operation. However, we soon recognized that this meningitis could have been caused by an inflammatory reaction to the obliterated *Echinococcus* antigens. Following the CT scan to confirm the absence of mass lesions, a cerebrospinal fluid (CSF) investigation was performed and revealed non-bacterial meningitis with monocyte-dominant pleocytosis of white blood cell count of 88/mm^3^ (97% monomorphonuclear, of which 1% were eosinophils), normal glucose level (53 mg/dl) and high protein level (82.5 mg/dl). CSF’s *Echinococcus* Western blot was strongly positive, indicating the presence of *Echinococcus* antigens in the CSF. While it remained unclear whether these antigens originated from live or obliterated *Echinococcus*, no suggestive signs of recurrence or progression were observable on contrast-enhanced brain and whole spine MRI. Unfortunately, none of the initial findings were sufficient to identify the cause of the meningitis (Table 1) [5, 9].

**Table 1:** Estimated results for meningitis by potential causes and our case findings

	**Recurrence of cerebral hydatidosis during albendazole therapy**	**Inflammatory reaction** **against the obliterated antigens**	**Our case**
Serologic tests			
Immunologic tests (e.g. Western blot, ELISA)	+	+ or −	Not performed
CSF			
Immunologic tests (e.g. Western blot, ELISA)	+	+ or -	+
Elevated eosinophil levels	+^a^ or –	+ (or −)^b^	−
MRI			
New contrast-enhanced lesions	+ or −	+ or −	−

^a^An elevated eosinophil level was observed at the recurrence of cerebral hydatidosis [9].

^b^Only one report has reported an elevated eosinophil level considerably due to the inflammatory reaction to obliterated *Echinococcus* antigen [5].

A decision was taken to initiate treatment based on the assumption that the meningitis was caused by an allergic reaction. Administration of prednisone [0.5 mg/kg/day (30 mg/day)] without intensifying chemotherapy was initiated based on these assumptions with careful MRI follow-up. The patient started to show significant clinical improvement soon after. The dose of prednisone was gradually reduced to the original dose prescribed for the treatment of rheumatoid arthritis during the coming 6 months. The administration of albendazole was terminated one year after the surgery (Fig. 1). The patient is free of symptoms and has no evidence of relapse on MRI as of 1.8 years after the initiation of treatment.

## Discussion

Signs suspicious of meningitis after removing CAHC could suggest a recurrence of CAH*.* Albendazole administration with occasional dose increases with or without adding another anthelmintic drug (praziquantel) is commonly performed to counteract possible disease recurrence [5, 10]. On the other hand, there are reports claiming amelioration in symptoms by administering steroids [5]. It is suggested that meningitis could sometimes be due to an inflammatory reaction and not a disease recurrence. In the present case, the patient’s symptoms appeared one month after initiating albendazole. They improved solely by adding steroids to the anthelmintic maintenance treatment, which is highly indicative of the cause being due to an inflammatory reaction. Moreover, it also supports our hypothesis that the one-month duration from the initiation of albendazole to the meningitis is consistent with the previous report [7]. To the best of our knowledge, this is the first report that successfully treated meningitis solely by adding prednisone after resection of CAHC.
